# Primitive and Definitive Neural Precursor Cells Are Present in Human Cerebral Organoids

**DOI:** 10.3390/ijms25126549

**Published:** 2024-06-14

**Authors:** Rehnuma Islam, Humna Noman, Ashkan Azimi, Ricky Siu, Vorapin Chinchalongporn, Carol Schuurmans, Cindi M. Morshead

**Affiliations:** 1 Institute of Medical Science, University of Toronto, 1 King’s College Circle, Toronto, ON M5S 3E1, Canada; 2 Department of Surgery, University of Toronto, 149 College Street, Toronto, ON M5T 1P5, Canada; 3 Sunnybrook Research Institute, 2075 Bayview Avenue, Toronto, ON M4N 3M5, Canada; 4 Department of Biochemistry, University of Toronto, 1 King’s College Circle, Toronto, ON M5S 3E1, Canada; 5 Department of Laboratory Medicine and Pathobiology, University of Toronto, 1 King’s College Circle, Toronto, ON M5S 3E1, Canada; 6 Donnelly Centre for Cellular and Biomolecular Research, University of Toronto, 160 College Street, Toronto, ON M5S 3E1, Canada; 7 Institute of Biomedical Engineering, University of Toronto, 164 College Street, Toronto, ON M5S 3G9, Canada

**Keywords:** human neural stem cells, cerebral organoids, neurospheres, caspase inhibitor, metformin

## Abstract

Activation of neural stem cells (NSCs) correlates with improved functional outcomes in mouse models of injury. In the murine brain, NSCs have been extensively characterized and comprise (1) primitive NSCs (pNSCs) and (2) definitive NSCs (dNSCs). pNSCs are the earliest cells in the NSC lineage giving rise to dNSCs in the embryonic and adult mouse brain. pNSCs are quiescent under baseline conditions and can be activated upon injury. Herein, we asked whether human pNSCs and dNSCs can be isolated during the maturation of human cerebral organoids (COs) and activated by drugs known to regulate mouse NSC behavior. We demonstrate that self-renewing, multipotent pNSC and dNSC populations are present in human COs and express genes previously characterized in mouse NSCs. The drug NWL283, an inhibitor of apoptosis, reduced cell death in COs but did not improve NSC survival. Metformin, a drug used to treat type II diabetes that is known to promote NSC activation in mice, was found to expand human NSC pools. Together, these findings are the first to identify and characterize human pNSCs, advancing our understanding of the human NSC lineage and highlighting drugs that enhance their activity.

## 1. Introduction

The developing and adult central nervous system (CNS) contains two populations of stem cells: definitive neural stem cells (dNSCs) and primitive neural stem cells (pNSCs). pNSCs are found as early as embryonic day (E) 5.5–7.5 and can be isolated in vitro to generate clonally derived colonies in the presence of leukemia inhibitory factor (LIF) [1,2]. pNSCs derived from the developing mouse embryo express low levels of the embryonic stem cell marker Oct4, as well as C-Kit, ErbB2, and the neural markers Sox1 and Nestin [1,2,3]. pNSCs are very rare cells that persist into adulthood along the neuraxis (brain and spinal cord), with a very long cell cycle time of 3–5 months in the adult mouse brain [1,4,5]. During development and in adult mice, lineage tracing has demonstrated that Oct4+ pNSCs generate neurons, astrocytes, and oligodendrocytes and give rise to dNSCs in the stem cell niche (the subependyma of the lateral ventricles) [6,7,8]. dNSCs are found later in development and can be isolated at E12.5 in the presence of epidermal growth factor and fibroblast growth factor to give rise to clonally derived colonies of neural precursor cells [5,6,7]. In adulthood, dNSCs are approximately ten times more abundant than pNSCs and have a shorter cell cycle time of 15–24 days [4,7,9,10]. Hence, the mammalian brain contains two populations of stem cells, pNSCs and dNSCs, that can self-renew and give rise to mature cells, which is important for brain repair and regenerative medicine.

Endogenous pNSCs and dNSCs are activated following CNS injury, such as stroke or spinal cord injury, but their response is not sufficient to promote neural repair [5,11]. Previous studies have correlated enhanced neural stem cell (NSC) activation with tissue repair and functional improvements in models of injury. For example, the administration of erythropoietin and/or growth factors after a traumatic brain injury increased the number of NSC-derived neurons and improved brain repair [12,13,14,15]. The administration of the drug cyclosporin enhanced dNSC survival, producing better functional outcomes after stroke [16,17]. Most interestingly, the type II diabetes medication metformin has been shown to promote neural stem and progenitor cell activation in mouse models of brain injury, which is correlated with functional recovery [18,19,20]. Numerous studies have correlated an enhanced dNSC response with improved functional outcomes in juvenile and adult mice [17,21,22]. pNSCs respond to injury and replenish the dNSC pool in the adult brain, providing evidence for their continued role in the adult injury response [4,5]. The role of pNSCs in promoting adult neural repair is an active area of research. These preclinical studies highlight the importance of understanding the fundamental biology of NSC pools and their injury response, with a view toward clinical translation. In line with this goal, dNSCs have been isolated from the human brain in the presence of epidermal growth factor [23]. The existence of pNSCs has not been examined in the human brain. We asked whether human dNSCs and pNSCs could be isolated during the maturation of human cerebral organoids (COs) and whether they can be drug-activated for neural repair.

Human cerebral organoids (COs) provide an opportunity to study human brain development, as they have been shown to recapitulate the early stages of embryonic development during their formation [24]. We hypothesize that pNSCs and dNSCs could be isolated from developing human COs, and their behavior could be modulated using drugs that have been proven efficacious in mice. Herein, we used early developing human COs to isolate and characterize pNSCs and dNSCs and tested their response to drugs and small molecules that have been shown to regulate mouse NSCs, specifically cell death and proliferation/survival.

## 2. Results

### 2.1. pNSCs and dNSCs Can Be Isolated from Human Cerebral Organoids

Similarly to the study of Lancaster et al., human COs were used to study early human brain development by recapitulating the formation of the laminar cortex [24,25,26,27]. We first confirmed that COs expressed markers of neurally committed genes, such as *FOXJ1*, *SOX10*, *MAP2*, *NES*, and *GFAP*, as previously demonstrated [28]. qPCR was performed on 10-day- to 110-day-old COs, and, as predicted, we observed reduced expression of the pluripotency gene *OCT4* and increased expression of the neurally committed gene *NES* (Supplemental Figure S1). qPCR revealed the upregulation of the transcripts for mature CNS cell types (astrocytes, *GFAP*; oligodendrocytes and oligodendrocyte precursors, *SOX10*; neurons, *MAP2*) with CO maturation in culture (Supplemental Figure S1).

**Figure 1:**
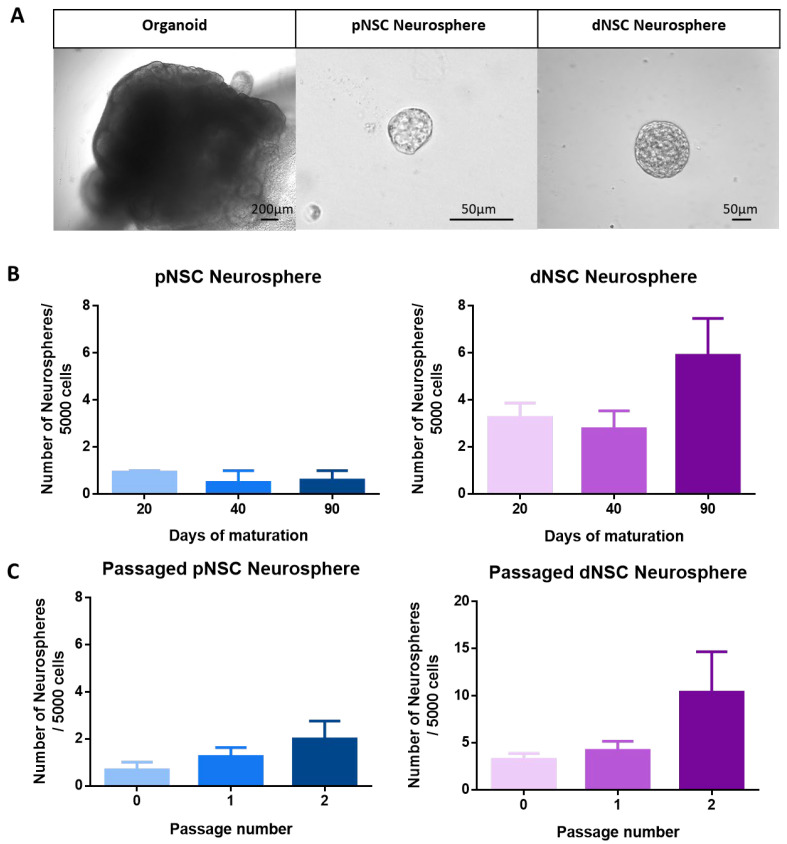
Human cerebral organoids contain pNSCs and dNSCs throughout their development: (**A**) representative images of a d20 CO, a pNSC neurosphere, and a dNSC neurosphere. (**B**) The numbers of neurospheres per 5000 cells plated from a single CO (d20, d40, and d90 of maturation). There was no significant difference in the numbers of pNSC and dNSC neurospheres grown across organoid maturation days. One-way ANOVA. *n* = 3 independent experiments; data represent the mean ± SEM. (**C**) Individual pNSCs and dNSCs from d20 and d40 COs were passaged in the same media conditions. The number of passaged neurospheres was not significantly different between passages. One-way ANOVA. *n* = 3 organoids per group; data represent the mean ± SEM.

The neurosphere assay is a colony-forming assay that has previously been used to identify pNSCs and dNSCs in cell culture [5]. To test whether pNSCs and dNSCs can be isolated in a model of human neural development, we examined 20-day to 90-day maturing COs for the presence of LIF-responsive (pNSCs) and EFH-responsive (epidermal growth factor, fibroblast growth factor, and heparin) (dNSCs) neurosphere-forming cells. Individual COs from day 20 (d20), d40, and d90 were collected, dissociated, and plated as 5000 cells per well in LIF or EFH conditions, and the numbers of neurospheres were counted after 7 days in vitro (Figure 1A,B). Single cells derived from COs at all maturation stages grew to form neurospheres in both LIF and EFH conditions, and the number of neurospheres was not significantly different across maturation stages (Figure 1B). Similarly to what was observed in mice [5], human dNSC neurospheres were larger in size (>80 µm diameter), and human pNSC neurospheres were smaller (~50 µm diameter) (Figure 1A,B). Irrespective of the age of the COs, pNSCs comprised ≤ 0.02% of plated cells, and dNSCs were approximately five times more abundant than pNSCs (0.1% of plated cells). Hence, pNSCs and dNSCs are present in developing human-derived COs and persist throughout CO maturation.

A cardinal property of stem cells is their ability to self-renew [2]. To assess the self-renewal of human pNSCs and dNSCs, neurospheres derived from d20 to d40 COs were bulk passaged as single cells into their respective media, and the number of newly formed neurospheres was counted. All passaged human dNSC and pNSC neurospheres gave rise to secondary and tertiary neurospheres (Figure 1C). While we observed a trend towards an increase in the number of passaged neurospheres with continued passaging, the expansion was not significant (Figure 1C). These results demonstrate that human dNSCs and pNSCs can self-renew over multiple passages.

### 2.2. Human pNSC and dNSC Neurospheres Have Distinct Genetic Expression Profiles

**Figure 2:**
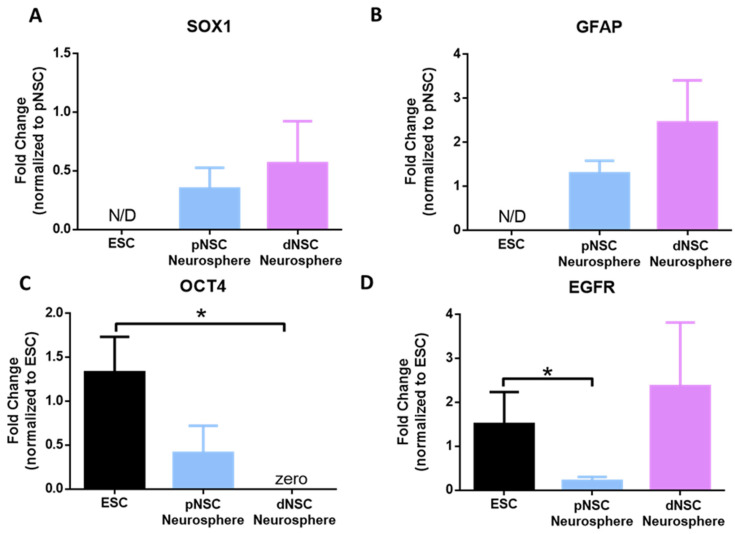
Gene expression analysis of human NSCs derived from cerebral organoids. qPCR was performed on ESCs and on dNSC and pNSC neurospheres. (**A**) pNSC and dNSC neurospheres express *SOX1*, a marker of neural commitment. (**B**) Both pNSC and dNSC neurospheres express *GFAP*. (**C**) ESCs and pNSCs express the pluripotency gene *OCT4*. (**D**) pNSC neurospheres express low levels of *EGFR* compared to ESCs and dNSCs. *n* = 3 organoids per group. * *p* < 0.05, mean ± SEM. N/D = not detected.

To confirm the unique identities of human pNSCs and dNSCs within COs, we grew neurospheres from d20 COs and collected them for gene expression analysis, probing for known genes expressed in mouse pNSCs and dNSCs. Previous work has demonstrated that pNSCs derived from mouse embryonic stem cells (ESCs) and the developing mouse brain are neurally committed [1,2]. We used qPCR to probe for the expression of the neural commitment gene *SOX1*. As shown in Figure 2A, both pNSC and dNSC neurospheres expressed *SOX1*, which was not observed in human ESCs, indicating that human pNSCs and dNSCs have a neural cell identity.

In the developing and postnatal mouse brain, dNSCs express *GFAP*, as do postnatally derived pNSCs [9]. Herein, we found that human pNSC and dNSC neurospheres from early (d20) COs expressed *GFAP* (Figure 2B). The difference between dNSC and pNSCs in mice is the expression of *OCT4*, as *OCT4* expression is unique to pNSCs through development and postnatally [4,5]. Consistently with the differential gene expression in the NSC pools, we found that human pNSCs expressed *OCT4*, while dNSCs significantly lacked expression (0.0015-fold, *p* < 0.05) when compared with human ESCs (Figure 2C). Further, EGF receptor (*EGFR*) was expressed in ESCs and dNSCs, with significantly lower expression in pNSCs (0.2-fold, *p* < 0.05) (Figure 2D) [29,30]. Taken together, these findings reveal that dNSCs and pNSCs isolated from human COs are distinct populations of NSCs with similar characteristics to those of NSCs isolated from developing and postnatal mice.

### 2.3. Human pNSC and dNSC Neurospheres Are Multipotent

**Figure 3:**
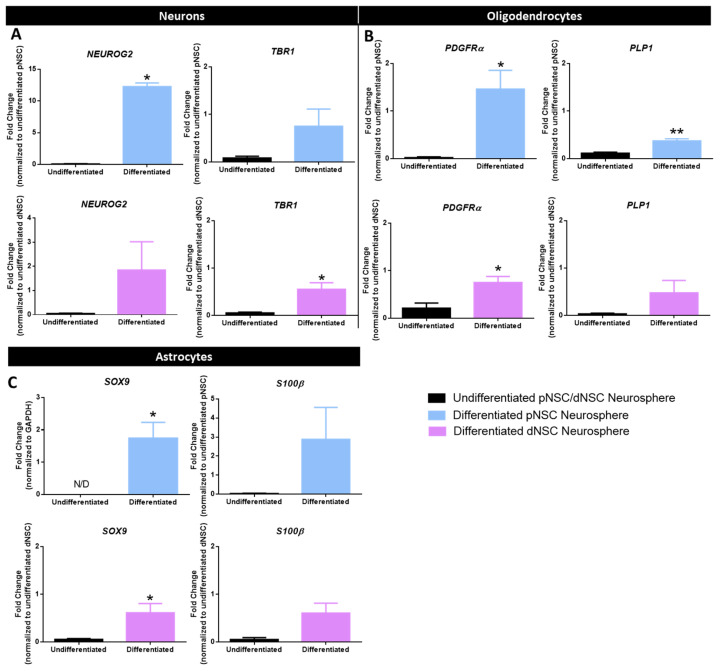
Human dNSC- and pNSC-derived neurospheres are multipotent. (**A**–**C**) Differentiated pNSC and dNSC neurospheres upregulated the expression of neuronal genes (*NEUROG2*, *TBR1*) (**A**), oligodendrocyte genes (*PDGFRα*, *PLP1*) (**B**), and astrocyte genes (*SOX9*, *S100β*) (**C**). Student’s *T*-test, * *p* < 0.05, ** *p* < 0.001. *n* = 3 organoids per group from which neurospheres were derived, mean ± SEM. N/D = not detected.

To demonstrate the multipotentiality of CO-derived NSC colonies, we performed qPCR for markers of neural phenotypes: intermediate neuronal progenitors (*NEUROG2*, a proneural gene turned off upon differentiation), neurons (*TBR1*, a cortical neuron marker), oligodendrocyte lineage (*PDGFRα*, an oligodendrocyte progenitor cell marker, and *PLP1*, a mature oligodendrocyte marker), and astrocytes (*SOX9*, a pan-astrocyte marker, and *S100β*, a mature astrocyte marker) from differentiated neurospheres. CO-derived pNSC and dNSC neurospheres were differentiated for 7 days, and the cells were collected for qPCR. We compared the expression of differentiated markers between undifferentiated and differentiated cells from pNSCs and dNSCs. As shown in Figure 3A–C, all differentiation genes were either absent or minimally expressed (0.01- to 0.4-fold) in undifferentiated neurospheres. Differentiated pNSCs expressed significantly more *NEUROG2*, *PDGFRα*, *PLP1*, and *SOX9* (*p* < 0.05) (Figure 3A–C). Differentiated dNSCs revealed significantly greater expression of *TBR1*, *PDGFRα*, and *SOX9* (*p* < 0.05) (Figure 3A–C). These findings reveal the multipotentiality of human pNSC- and dNSC-derived colonies.

### 2.4. Human CO-Derived Neural Precursor Cells Are Responsive to Pro-Survival and Proliferation Factors Identified in Mice

**Figure 4:**
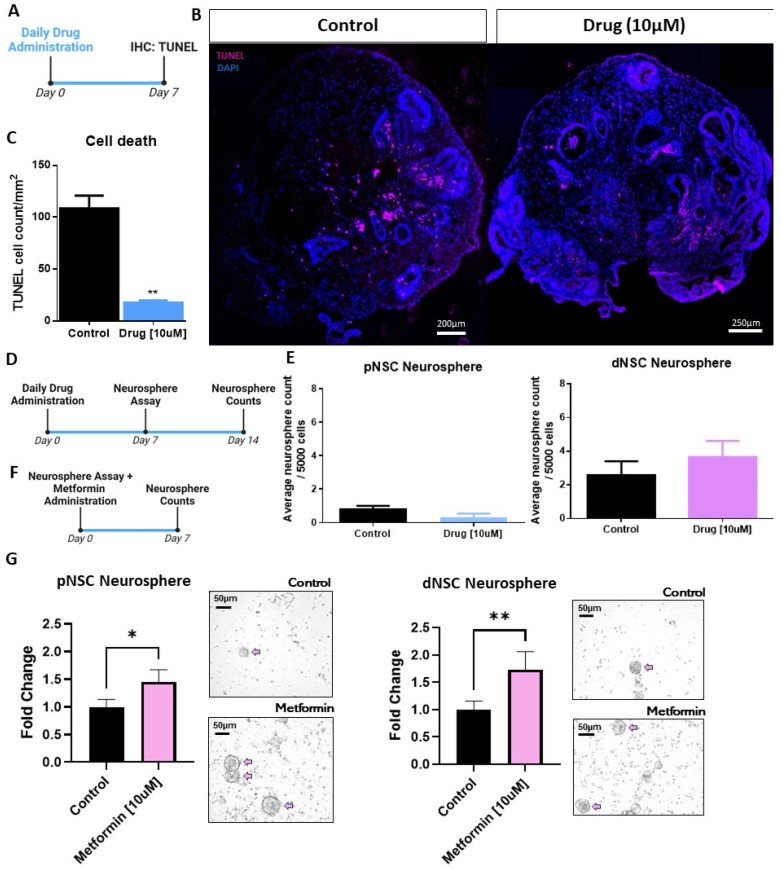
Caspase-3 and -7 inhibition prevents cell death and metformin NSC activation in organoids. (**A**) Experimental paradigm: Organoids were placed in media containing 10 µM NWL283 for 7 days and collected for TUNEL labeling. (**B**) Organoids stained with TUNEL (red) and DAPI (blue). (**C**) Quantification of TUNEL-positive cells reveals reduced cell death in drug-treated COs. Student’s *T*-test, ** *p* < 0.01. *n* = 3 organoids per group; data represent the mean ± SEM. (**D**) Experimental paradigm examining the number of neurospheres that formed from COs grown in the presence or absence of the drug for 7 days, followed by dissociation and plating in pNSC- or dNSC-neurosphere-forming conditions in the presence or absence of the drug. (**E**) Quantification reveals no difference in the number of pNSCs or dNSCs irrespective of the drug treatment. Student’s *T*-test. *n* = 3 organoids per group; data represent the mean ± SEM. (**F**) Experimental paradigm with COs dissociated and plated in neurosphere-forming conditions, with or without 10 µM metformin, for 7 days. (**G**) Metformin significantly increased the numbers of pNSCs and dNSCs. Bright-field images of neurospheres (pink arrows). Paired *T*-test, * *p* < 0.05. ** *p* < 0.01. *n* = 7 organoids per group; data represent the mean ± SEM.

Maturing COs are known to develop a necrotic core with cells undergoing apoptosis in the periphery [31]. A proprietary drug, NWL283, has been shown to inhibit caspase-3 and -7 mediated apoptosis of mouse dNSCs both in vitro and in vivo [22]. Here, we asked if human neural precursor cells were responsive to NWL283 by exposing d20 COs to NWL283 for 7 days followed by TUNEL labeling to assess cell death (Figure 4A,B). As shown in Figure 4B,C we observed a significant >5-fold reduction in the number of TUNEL-positive cells in COs exposed to NWL283 compared to the untreated sample (*p* < 0.01). We next asked if the NSCs within COs were responsive to NWL283; d20 COs exposed to 10 µM NWL283 for 7 days were collected and dissociated into single cells for plating in EFH (dNSC conditions) or LIF (pNSC conditions), with or without NWL283, for an additional 7 days (Figure 4D). The numbers of dNSC and pNSC neurospheres were unchanged irrespective of the NWL283 treatment (Figure 4E). Together, these data suggest that human neural precursor cell survival can be enhanced by inhibiting caspase-3- and -7-dependent apoptosis.

We next asked whether human NSCs were responsive to the type II diabetes drug metformin, which has been shown to expand the size of the NSC pool in mouse models [18,20,21]. To assess whether human NSC populations respond to metformin treatment, we performed the neurosphere assay with d60–90 COs in the presence or absence of 10 µM metformin (Figure 4F,G). We found a significant expansion in pNSC (1.5-fold increase, *p* < 0.05) and dNSC (1.6-fold increase, *p* < 0.01) neurosphere formation in the presence of metformin (Figure 4G). Taken together, these findings reveal that human NSCs comprise distinct pNSC and dNSC pools, and their kinetics can be regulated by exogenous factors, similarly to the NSCs that comprise the neural stem cell lineage in mice.

## 3. Discussion

This study is the first to identify and characterize human LIF-responsive pNSCs from human COs. We showed that human pNSCs and dNSCs are neurally committed, multipotent, and self-renewing stem cells. They have similar genetic signatures to those that were previously characterized in the mouse neuraxis [1]. These findings have important implications for our current understanding of the human NSC lineage in development.

We observed several similarities between mouse and human pNSC and dNSC colonies, including their size, frequency, and gene expression profiles. One difference is that the passaging of human neurospheres did not result in an expansion of the NSC pool. Passaged mouse primary dNSCs, but not primary pNSCs, expand in number, revealing symmetric divisions of NSCs in neurosphere colonies [32]. The lack of expansion of human dNSCs suggests asymmetric cell division or a lack of cell division in neurospheres. In development and within organoids, the outer radial glia asymmetrically divide, producing more intermediate progenitor cells that generate cortical neurons [24,33]. Notably, we found that human pNSCs and dNSCs from early (20d) COs expressed GFAP, which is expressed in radial glial cells with NSC properties during development. Thus, our findings suggest that early organoid-derived pNSCs and dNSCs divide asymmetrically to generate progeny without expanding in number.

Human pNSC- and dNSC-derived cells can differentiate into all three neural cell types—neurons, oligodendrocytes, and astrocytes—thereby revealing the cardinal property of multipotentiality. NSC differentiation induced the expression of the early neuronal and glial differentiation genes *NEUROG2*, *PDGFRα*, and *SOX9*. To a lesser extent, there was gene expression of the mature neuronal gene *TBR1* and the mature oligodendrocyte gene *PLP1*. The gene expression profiles suggest that pNSC colonies gave rise to more progenitors while dNSCs differentiated into more mature cells from the neuronal and oligodendrocyte lineages. We cannot rule out the possibility that human ESC-derived NSC colonies require longer times to differentiate into mature phenotypes, but the distinct differentiation profiles of human-derived pNSC and dNSC colonies observed are similar to what has been reported in mouse NSCs [9,17].

There is a correlation between neural precursor cell activation and neural repair [34]. The activation of NSCs and their progeny has been demonstrated using a variety of stimuli, including drugs, growth factors, cell survival factors, and electrical stimulation [17,18,35,36,37]. It is not clear whether human NSCs are similarly responsive to the activation strategies that have been demonstrated in rodent models. Herein, we did not observe the pro-survival effects of caspase inhibition on human dNSCs, as was reported in mice, which may suggest that human NSCs undergo cell death through non-apoptotic pathways, such as autophagy, necrosis, or necroptosis [22,31,38,39]. Consistently with previous studies showing that cell death is prominent in developing COs, we observed less TUNEL labeling in COs grown in the presence of the apoptotic inhibitor.

One of the limitations of using COs to test the effects of drugs is the lack of vascularization and other neural cells [40,41]. In our studies with NWL283, it is noteworthy that previous studies examining the role of caspase-3 in the CNS suggest that it may have unconventional roles related to astrogliosis without directly regulating astrocyte death [25,42]. Islam et al. found that the administration of NWL283 to the post-stroke adult brain reduced microglial activation and could not rule out a direct effect of caspase-3 inhibition on microglia, which are absent in developing COs [43]. Co-culturing microglia with maturing COs is an important step to further study the effect of drug treatment on microglia-mediated cell death [44]. Most interesting, we demonstrated the pro-survival effects of caspase-3 inhibition on developing COs comprised of neural precursor cells independent of endothelial cells and microglia.

Metformin has been shown to have pleiotropic effects in the mammalian brain. Previous studies have characterized both the effect and mechanism of action of metformin on mouse NSCs [18,19,20,21,45,46]. Not only has metformin been shown to promote neurogenesis and oligogenesis in vitro, but 1 week of metformin treatment led to functional recovery in a neonatal model of brain injury known as hypoxic ischemia [18,47,48,49]. The recovery was correlated with the expansion of the size of the NSC pool and migration of progenitors to the injured parenchyma [18]. In the same model of hypoxic ischemia, metformin treatment resulted in a reduction in inflammation as quantified by the cellular morphology of microglia, while no differences were observed for astrocyte populations [50]. COs, due to their lack of microglia, provide us with a unique opportunity to assess metformin’s direct effects on neural cells. Overall, our findings have implications for understanding the role of NSCs in neural repair [51,52], and future studies involving microglia-containing COs could provide further insights into their role in metformin-mediated neural repair.

## 4. Materials and Methods

### 4.1. Embryonic Stem Cell Culture

Human H1 ESCs (WiCell, Madison, WI, USA) were grown on Geltrex-coated (A1413302 Thermo Fisher Scientific, Waltham, MA, USA) tissue culture plates in mTESR1 media and replaced daily (85850 STEMCELL Technologies, Vancouver, BC, Canada) as previously described [53]. At 70–80% cell density, cells were lifted using a gentle cell dissociation reagent (07174 STEMCELL Technologies, Vancouver, BC, Canada) and either passaged 1:6 onto six-well plates (CLS3471-24EA Millipore Sigma, Oakville, ON, Canada) or used for organoid culturing.

### 4.2. Cerebral Organoid Culture

Human H1 ESCs were cultured as organoids according to the procedures in the STEMdiff cerebral organoid kit (008570, STEMCELL Technologies, Vancouver, BC, Canada) [54]. Briefly, ESCs were plated at a density of 90,000 cells/µL of Embryoid Body (EB) formation media (008570, STEMCELL Technologies, Vancouver, BC, Canada) in 96-well U-bottom ultra-low-attachment plates (7007, Corning, Corning, NY, USA). Formation media were added every other day for 4 days, followed by the transfer of EBs into induction media (008570, STEMCELL Technologies, Vancouver, BC, Canada) in 24-well ultra-low-attachment plates (3473, Corning, Corning, NY, USA) for two days. The EBs were then embedded in Matrigel (354277, Corning, Corning, NY, USA) and cultured in six-well ultra-low-attachment plates (3471, Corning, Corning, NY, USA) containing expansion media (008570, STEMCELL Technologies, Vancouver, BC, Canada) for two days and then replaced with organoid maturation media (008570, STEMCELL Technologies, Vancouver, BC, Canada). Cultures were placed on an orbital shaker at 70 rpm or on a spinning mini-bioreactor. Organoid maturation was changed every 3–4 days thereafter until the time of organoid collection (days 20, 40, and 90). A modified dual-SMAD inhibitor protocol was also used to grow organoids as previously described [55,56]. Following similar procedures to those above, this protocol included the addition of 2 µM dorsomorphine (inhibitor of BMP type I receptors) (72102, STEMCELL Technologies, Vancouver, BC, Canada) and 2 µM A83-01 (inhibitor of TGFβ type I receptors) (72022, STEMCELL Technologies, Vancouver, BC, Canada) on days 1 and 3. On day 5, 1 µM SB431542 (an ALK4, ALK5, and ALK7 inhibitor) (72234, STEMCELL Technologies, Vancouver, BC, Canada) and 1 µM CHIR99021 (GSK3β inhibitor) (72054, STEMCELL Technologies, Vancouver, BC, Canada) were also added to the induction media. EBs were then embedded into Matrigel on that day and placed in expansion media supplemented with 1 µM SB431542 and 1 µM CHIR99021. On the 13th day, each well of a 12-well mini spinning bioreactor was filled with individual EBs along with maturation media from the STEMdiff kit. Starting from the 30th day, the maturation media were enhanced with extracellular matrix (ECM) proteins by incorporating 1% (*v*/*v*) Matrigel containing human recombinant brain-derived neurotrophic factor (AF-450-02, Peprotech, Cranbury, NJ, USA). Organoids were allowed to develop in maturation media until the specified endpoints.

### 4.3. Neurosphere Assay

COs were dissociated in 1 mL of NeuroCult-XF proliferation medium (05761, STEMCELL Technologies, Vancouver, BC, Canada) using an 18G x1½ blunt-fill needle (305180, BD, Franklin Lakes, NJ, USA) and a 3 mL syringe. The homogenate was passed through a 40 µm sterile filter, and the filtrate was collected for single-cell counting. Based on previous culture conditions to isolate human and mouse NSC populations [8,23,57], cells were plated at a density of 10 cells/µL in NeuroCult-XF proliferation media supplemented with epidermal growth factor (20 ng/mL) (AF-100-15, Peprotech, Cranbury, NJ, USA), fibroblast growth factor (30 ng/mL) (AF-100-18B, Peprotech, Cranbury, NJ, USA), and 100 µg/mL heparin (H3149-100KU, Millipore Sigma, Oakville, ON, Canada) or with human leukemia inhibitory factor (LIF, 10–50 ng/mL) (LIF1005, Millipore Sigma, Oakville, ON, Canada). The numbers of colonies (termed neurospheres) in EFH and LIF were counted 8 days after plating.

To assess the differentiation potential of NSC-derived colonies, free-floating neurospheres were differentiated in NeuroCult differentiation media (05752, STEMCELL Technologies, Vancouver, BC, Canada) for 7 days and collected for qPCR.

### 4.4. NWL283 Administration

NWL283 (New World Laboratories, Laval, QB, Canada) is a proprietary caspase-3 and -7 inhibitor. NWL283 was diluted in ddH2O to obtain a 1000 µM stock concentration and a final concentration of 10 µM in maturation media (008570, STEMCELL Technologies, Vancouver, BC, Canada) and replaced daily in organoid cultures for 7 days, at which time the organoids were collected.

### 4.5. Metformin Administration

Metformin (1,1-dimethylbiguanide hydrochloride, D150959, Millipore Sigma, Oakville, ON, Canada) was dissolved in NeuroCult-XF proliferation medium at a stock concentration of 1000 µM. It was then dissolved further in human LIF- and EFH-specific supplemented media (described above) to a final concentration of 10 µM, which is the optimized concentration for mouse NSC expansion [21].

### 4.6. TUNEL Labeling

COs were stored in 30% sucrose until cryosectioning (−20 °C). Then, 10 µm thick sections were placed on SuperFrost slides (1255015, ThermoFisher Scientific, Waltham, MA, USA). TUNEL labeling was performed using the Click-IT Plus TUNEL Assay Detection (C10619, ThermoFisher Scientific, Waltham, MA, USA) and Alexa Fluor647 kits (A10277, ThermoFisher Scientific, Waltham, MA, USA) as per the manufacturers’ instructions. Sections were stained for DAPI using VectaShield (H-1200, Vector Laboratories, Newark, CA, USA), cover-slipped, imaged, and counted using the QuPath detection software version 0.2.3 [58], and confirmed manually by a blinded observer. A minimum of 3 sections per CO were imaged, and cell counts were performed on the entire CO section.

### 4.7. qPCR

Neurospheres were grown in EFH (dNSCs) or LIF (pNSCs) and subsequently collected, plated, and differentiated for 7 days in Neurocult differentiation media (05752, STEMCELL Technologies, Vancouver BC, Canada). For analysis, neurospheres or differentiated cells were stored in their respective culture media at −80 °C for up to three months. For controls, RNA was extracted from cultured ESCs using the RNAeasy Plus Mini Kit (74104, Qiagen, Hilden, Germany) by following the manufacturer’s instructions. cDNA was generated from 10 ng of ESC RNA using a cDNA synthesis kit (1725035, Bio-Rad; Mississauga ON, Canada) as per the manufacturer’s instructions. cDNA from 5–20 undifferentiated and differentiated neurospheres was generated and amplified from whole-cell RNA using the SMART-Seq v4 Ultra-Low-Input RNA Kit for Sequencing (634888, Takara Bio, San Jose, CA, USA) following the manufacturer’s instructions. The PCR Primer II A recognizes the SMART-Seq v4 Oligonucleotide and primes the cDNA for amplification by DNA polymerase, with each cycle totaling 14 cycles. QPCR was run using 100–150 ng/µL of cDNA, SsoAdvanced™ Universal SYBR^®^ Green Supermix (1725271, Bio-Rad; Mississauga, ON, Canada), and a QuantStudio (™) 6 Flex System (Applied Biosystems, Waltham, MA, USA) for 40 cycles.

Verified primers were obtained for human *OCT4 (POU5F1)*, *SOX1*, *EGFR*, *GFAP*, *SOX 9*, *SOX10*, *FOXJ1*, *NESTIN*, *PDGFR*, *S100β*, *TBR1*, *NEUROG2*, *PLP1*, and *GAPDH* (Bio-Rad, Mississauga, ON, Canada) (Supplemental Table S1).

### 4.8. Statistical Analysis

Statistical analysis was conducted using GraphPAD Prism V6, and the analysis was conducted with ANOVA, Tukey’s post hoc test for multi-group comparisons, and Student’s *T*-test, as well as the Welch or Mann–Whitney post hoc tests for two-group comparisons. Outliers were removed using Grubb’s test. All data are represented as the mean ± SEM. Significant differences are considered at *p* < 0.05.

## 5. Conclusions

This is the first study to identify a pNSC population that can be isolated from developing human organoids, demonstrating similar characteristics to those that have been extensively studied in mice. We have shown that human dNSCs and pNSCs can be cultured from COs on different maturation days using conditions known to promote in vitro mouse NSC cultures. Interestingly, human NSCs grown from COs maintain an asymmetric mode of division, producing predominantly progenitor cells. Caspase-3 inhibitors affect the survival of other cells without affecting NSC survival within cerebral organoids, while metformin enhances NSC activation. Our study demonstrates that COs possess similar populations of NSCs to those that were previously characterized in mice and provide a tool to study neural repair and the neural lineage in human development. Cross-species therapeutic testing is, therefore, pivotal in translational research.

## Data Availability

Data are contained within the article and Supplementary Materials. Additional data presented in this study are available from the corresponding author upon request.

## Supplementary Materials

The supporting information can be downloaded at: https://www.mdpi.com/article/10.3390/ijms25126549/s1.
