# Supplementary figures and images for: Primitive and Definitive Neural Precursor Cells Are Present in Human Cerebral Organoids
Source: Int J Mol Sci. 2024 Jun 14;25(12):6549. doi: 10.3390/ijms25126549 (PMC11203442; doi:10.3390/ijms25126549)

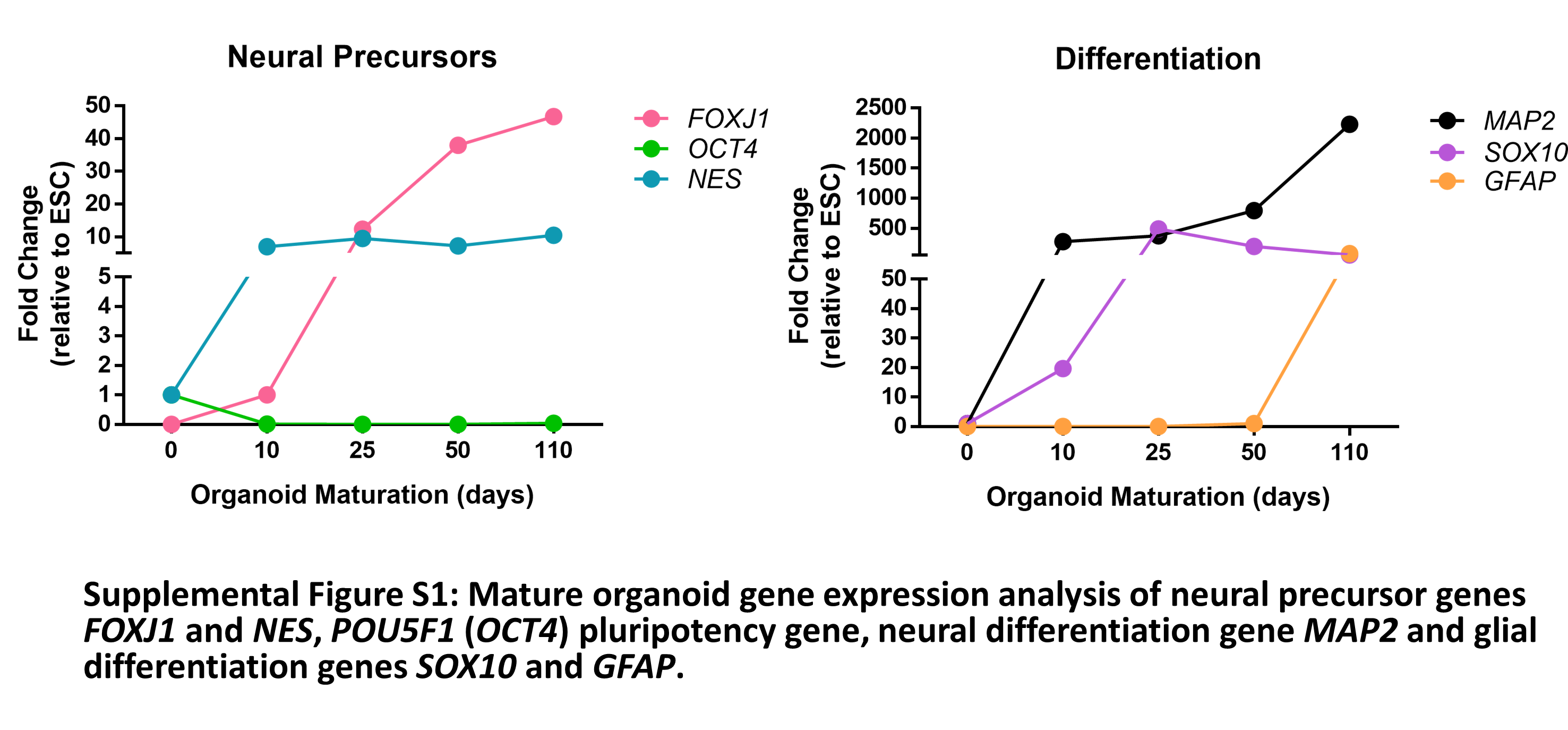

Supplement: Supplementary file 1 [file ijms-25-06549-s001.zip › Supplemental Figure S1.TIF]

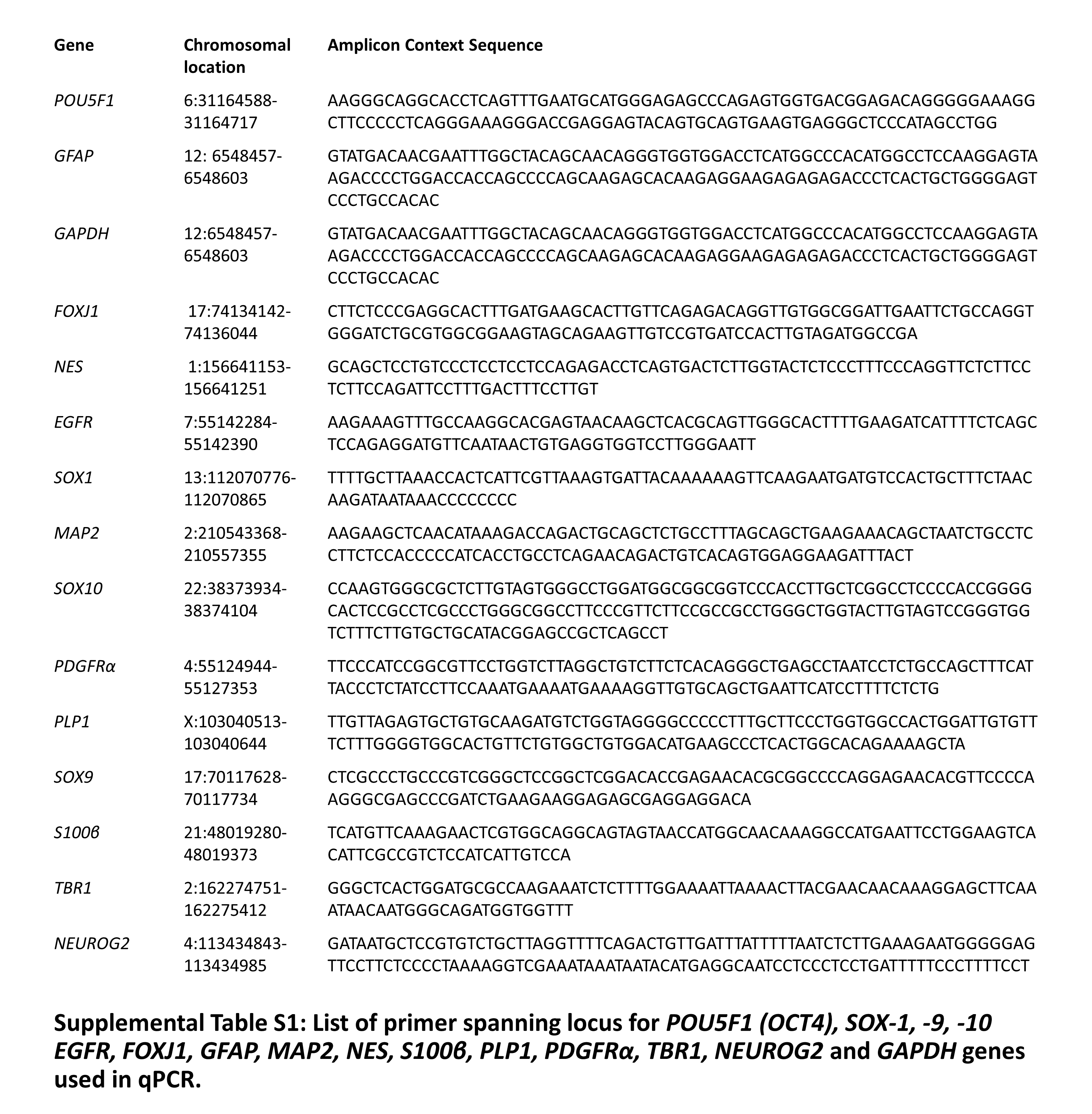

Supplement: Supplementary file 1 [file ijms-25-06549-s001.zip › Supplemental Table S1.TIF]
